# Medical Society of the State of New York

**Published:** 1911-07

**Authors:** 


### Medical Society of the State of New York

ABSTRACT from Report of the President, Dr. Charles W. Stover, of Amsterdam, to the House of Delegates at the one hundred and fifth annual meeting held at Albany, April 18 and 19, 1911.

"The services of William Warren Potter, editor of the BUFFALO MEDICAL JOURNAL, and as a member of the New York State Board of Medical Examiners and of the National organisation likewise, were marked by loyalty to the medical profession and its higher advancement. As secretary of the American Association of Obstetricians and Gynecologists, his business methods and careful editing of its annual proceedings will never be surpassed. His social and conversational characteristics were admirably blended and strongly tinted by his experiences as surgeon during the civil war.

